# Recovery From a Forward Falling Slip: Measurement of Dynamic Stability and Strength Requirements Using a Split-Belt Instrumented Treadmill

**DOI:** 10.3389/fspor.2020.00082

**Published:** 2020-07-21

**Authors:** Héloïse Debelle, Carla Harkness-Armstrong, Kathryn Hadwin, Constantinos N. Maganaris, Thomas D. O'Brien

**Affiliations:** Research Institute for Sport and Exercise Sciences, Liverpool John Moores University, Liverpool, United Kingdom

**Keywords:** slip, fall, instrumented treadmill, dynamic stability, joint moment, balance recovery

## Abstract

**Aim:** Falls commonly occur from trips and slips while walking. Recovery strategies from trips and backward falling slips have been extensively studied. However, until recently, forward falling slips (FFSs) have been considered less dangerous and have been understudied. This study aimed first to create an application to realistically simulate FFSs using a split-belt instrumented treadmill and then to understand the biomechanical requirements for young adults to recover from an FFS.

**Methods:** We developed a semi-automatic custom-made application on D-Flow that triggered FFSs by briefly and unexpectedly increasing the speed (*a* = 5 m·s^−2^) of the right belt during stance. To validate the protocol, we tested against criteria defined for an ecologically and experimentally valid FFS: unexpected occurrence of the slip, increased foot velocity, forward loss of balance during the slip and consistent perturbation timing. We evaluated the recovery strategies of 17 young adults by measuring dynamic stability, joint moments and ground reaction force (GRF) vector angles before, during and on 15 steps following the FFS.

**Results:** The application successfully triggered FFSs, according to the criteria we defined. Participants' balance returned to normal for a minimum of three consecutive steps in 10.9 (7.0) steps. Recovery from the FFSs was characterised by larger hip flexor and knee extensor moments to support the centre of mass during the slip, and a longer first recovery step with large hip extensor moments to arrest the fall followed by large knee extensor moments to raise and advance the centre of mass into the next step (*p* < 0.001 compared with normal gait). Subsequent steps progressively returned to normal.

**Conclusion:** This is the first study to experimentally simulate FFSs meeting the aforementioned criteria, and to measure their effects on the dynamic balance and kinetic parameters. The split-belt instrumented treadmill proved a promising tool to better study the mechanisms of falls and recovery. The required large hip and knee joint moments generally agree with findings on trips and backward falling slips and provide an indication of the functional capacities that should be targeted in fall-prevention interventions. These findings should be used to better understand and target the mechanisms of balance loss and falls in older adults following FFSs.

## Introduction

The prevalence of falls among the general population goes from 1:5 to 1:2 and increases with age (Talbot et al., 2005). Falls mostly result from a trip or a slip while walking (Berg et al., 1997) and can lead to serious injuries, hospitalisation, or momentary or permanent loss of autonomy. Therefore, to reduce the social and economic cost of falls, we must understand the mechanisms by which they occur and how we can prevent them. It may be possible to reduce the likelihood of the initial balance perturbation, for example, by improving the environment especially for people at a high risk of falling (Nikolaus and Bach, 2003; Lord et al., 2007), but when a postural perturbation cannot be avoided, balance must be recovered to prevent it from becoming a fall. To this end, it is necessary to study fall recovery strategies, with a view to developing comprehensive interventions to prevent injurious falls.

Studies investigating recovery from a trip (i.e., a perturbation in which the foot is suddenly arrested during the swing phase by contact with an object) have found that the recovery strategy depends on the perturbation timing (Eng et al., 1994) and that successful recovery in young adults is achieved mainly by developing large hip, knee, and ankle extensor moments during stance of the contralateral and ipsilateral limbs (Pijnappels et al., 2004; King et al., 2019). These kinetic responses control the body's angular momentum and provide time to enlarge the base of support (BoS) size and to better control the centre of mass (CoM) position and velocity (Pijnappels et al., 2004; Suptitz et al., 2013).

Similarly to trips, timing of a slip (i.e., a perturbation in which the foot velocity is increased anteriorly or posteriorly during the stance phase) also determines the outcome of the perturbation. Slips happen when the required coefficient of friction [ratio of horizontal to vertical ground reaction force (GRF) magnitude] exceeds the available coefficient of friction at the interface between the shoe and the floor (Redfern et al., 2001). Slips mainly occur either during loading of stance when the foot slides forward, inducing a backward fall, or during push-off when the foot slides backward causing a forward fall. When recovering from backward falling slips (BFSs), previous studies have shown that participants initially rely on increased hip extensor and knee flexor moments during loading of the slipping limb (Cham and Redfern, 2001), on large knee extensor and ankle dorsiflexor moments from mid to late stance (Liu and Lockhart, 2009) and on larger hip and knee flexor and extensor moments in the first recovery step (Yoo et al., 2019).

The mechanisms and kinetic requirements of successful recovery from forward falling slips (FFSs) are not yet as well-understood. This could be because FFSs had been considered less risky, as they happen at the end of stance when the body weight is being transferred to the contralateral foot (Strandberg and Lanshammar, 1981). However, a study conducted on contaminated oily floors found that FFSs occurred 2.5 times more frequently than BFSs (Nagano et al., 2013). Further, although Myung (2003) observed 40% more BFSs than FFSs while walking on oily surfaces, the number of FFSs that had to be stopped by the safety harness, indicating they would have led to an actual fall event, was double that of BFSs. Although the ecological relevance and recovery mechanics of BFSs are well-understood (Cham and Redfern, 2001; Redfern et al., 2001; Bhatt et al., 2006b; Yoo et al., 2019), it seems that the frequency and dangerousness of FFSs have been largely underestimated, and as a result, fall-prevention research has neglected to also study the mechanics of FFSs.

Previous studies on FFSs have used oily surfaces (Myung, 2003; Nagano et al., 2013) and shoe apparatus (Rasmussen and Hunt, 2019). Although these methods successfully triggered FFSs, they are each limited in how they enhance our understanding of slips and recovery strategies in some way. Studies conducted on oily surfaces are constrained by the location of the contaminated surface on the walkway, which gives visual cues to participants who may adapt their gait in anticipation. They also do not allow the study of post-slip recovery strategies and kinetics because of the contaminant presence at least on one sole or even on the floor. Shoes apparatus were found to be promising in delivering multiple timing perturbations, but to study kinetics, they remain limited to locations of force plates and, therefore, do not provide balance recovery analysis of multiple steps following the perturbation. This is important because it has been shown that older adults need several steps to recover (Suptitz et al., 2013).

Alternatively, split-belt treadmills have been used to simulate FFSs by increasing the posterior velocity of one belt, causing a forward falling loss of balance (Lurie et al., 2013; Ilmane et al., 2015; Sloot et al., 2015; Madehkhaksar et al., 2018; Roeles et al., 2018; Gholizadeh et al., 2019). These studies provided insights on balance recovery, kinematics, muscular activity and the use of treadmills to build fall-prevention interventions. However, they did not faithfully replicate the characteristics of an actual slip. Specifically, the timing of the perturbation was not always controlled (Lurie et al., 2013; Madehkhaksar et al., 2018), and the belt acceleration was sometimes triggered at heel strike (Roeles et al., 2018) or during the swing phase (Ilmane et al., 2015), which is too early to be representative of real-life FFSs. Like in recovery from a trip (Eng et al., 1994; Schillings et al., 2000), it is expected that timing is important in the adopted recovery strategy from a slip. Additionally, by triggering the perturbation during the swing phase or allowing perturbation familiarisation (Ilmane et al., 2015; Madehkhaksar et al., 2018), participants may have developed anticipatory responses to the perturbation or experienced a learning effect, respectively. Finally these studies did not provide a biomechanical analysis of the kinetic requirements of balance recovery following an ecologically valid FFS.

To study balance recovery strategies following an FFS, we need to develop a protocol that utilises the principle of accelerating a split-belt instrumented treadmill to allow biomechanical measurements of dynamic balance and recovery mechanisms with ecological and experimental validity and that offers realistic ecological validity. To the best of our knowledge, data from real (i.e., outside of lab-controlled environments) slips have never been reported. Therefore, the criteria by which ecological validity can be defined must be based on data from lab-induced slip-like perturbations, which themselves have not been fully validated in real conditions. Initially, the participant must lose control of their CoM as a result of the perturbation, which can be quantified using the margin of stability (MoS). The MoS was first introduced by Hof et al. (2005) and is now commonly used to measure balance in dynamic conditions. Briefly, it measures the distance between the extrapolated CoM (XCoM), which accounts for the position and velocity of the CoM, and the boundaries of the BoS, either anteriorly when evaluating forward falling gait perturbations like trips or FFSs (Suptitz et al., 2013; Roeles et al., 2018) or posteriorly when evaluating backward falling gait perturbations like BFSs (Bhatt and Pai, 2009). The effects on balance of forward falling perturbations remain unclear. Although studies conducted on trips found that the MoS of participants decreased on the first recovery steps (Suptitz et al., 2013; Epro et al., 2018a), data reported from FFS-like perturbations suggest that although highly variable (Madehkhaksar et al., 2018), the size of the MoS (averaged on multiple recovery steps) may not be significantly impacted by the slip (Madehkhaksar et al., 2018; Roeles et al., 2018). Contradictory results show that the position and velocity of the CoM are affected by a slip-like perturbation, with the CoM being positioned more anteriorly to the BoS than in normal conditions on the first recovery step following the perturbation (Ilmane et al., 2015). According to Nagano et al. (2013), FFSs are characterised by a large posterior toe sliding velocity during the slip (~1.6 ± ~1.0 m·s^−1^). Therefore, according to the available data, it appears that an ecologically valid FFS would be characterised by an unexpected and large posterior foot sliding velocity during the slip leading to forward loss of balance in the second half of stance and at least until heel strike of the contralateral leg. In trips, forward loss of balance remained evident in the first four recovery steps following the perturbation onset (Epro et al., 2018a). Finally, to be experimentally valid, the perturbation should be repeatable and allow measurements of kinematics and kinetics variables before, during and after the perturbation.

By increasing the velocity of a split-belt instrumented treadmill, we can now develop perturbation protocols to simulate slips. These can be triggered at any time point of stance and have the potential to meet the aforementioned criteria of ecologically and experimentally valid perturbations while also measuring the mechanics of fall avoidance and balance recovery.

The aims of this study were therefore to develop a realistic and consistent FFS simulation using a split-belt instrumented treadmill and then to use that protocol to identify the kinematics and kinetics requirements of fall avoidance and balance recovery.

## Materials and Methods

### Participants

Seventeen participants (eight males and nine females, age 25.2 ± 3.7 years, height 176.1 ± 8.1 cm, body mass 71.8 ± 10.1 kg) were recruited to test the FFS protocol and evaluate the effect of the FFSs on dynamic stability and recovery strategies. Participants had no self-reported recent history of lower limb musculoskeletal injuries or neural, musculoskeletal or balance disorder that could affect their balance or walking. During data collection, participants wore a full-body safety harness attached to a frame above the treadmill to prevent injuries should they fall.

The protocol was approved by the Liverpool John Moores University ethics committee, and written informed consent was obtained from each participant. All human testing procedures undertaken were conformed to the standards of the Declaration of Helsinki.

### Experimental Protocol

Participants walked on a dual-belt force plate instrumented treadmill (1,200 Hz, Motekforce Link, Amsterdam, Netherlands) at constant speed (1.2 m·s^−1^) for at least 5 min before data collection. Following this familiarisation period, kinetic and kinematic data were recorded during five gait cycles to quantify normative gait parameters (Normal). We then recorded kinetic and kinematic data two steps prior to the slip-simulating perturbation (Pre1 and Pre2), during the slip (Slip) and on at least 15 steps afterwards (Rec1 to Rec15).

A six-degrees-of-freedom full body marker set was used, and 68 retroreflective markers were tracked by 12 motion capture cameras (120 Hz, Vicon Motion Systems, Oxford, UK). Kinetic and kinematic data were filtered using a low-pass fourth-order Butterworth filter with a cut-off frequency of 8 Hz.

### Slipping Perturbation Protocol

A custom D-Flow application was developed to increase the right belt speed during stance in the posterior direction. This displaced the foot posteriorly to the CoM at a greater rate than during normal gait, thereby simulating an FFS.

#### Perturbation

##### Intensity

Pilot tests conducted at 3, 4, 5, 6, and 7 m·s^−2^ suggested that until 4 m·s^−2^, the perturbation was not challenging enough and that from 6 m·s^−2^, some participants had great difficulties to recover and spontaneously used the handrails to prevent a fall. As the aim of the study was to understand the recovery mechanisms from realistically challenging perturbations, the acceleration was set at 5 m·s^−2^ to increase and then decrease the velocity of the right treadmill belt.

##### Duration

**Figure 1:**
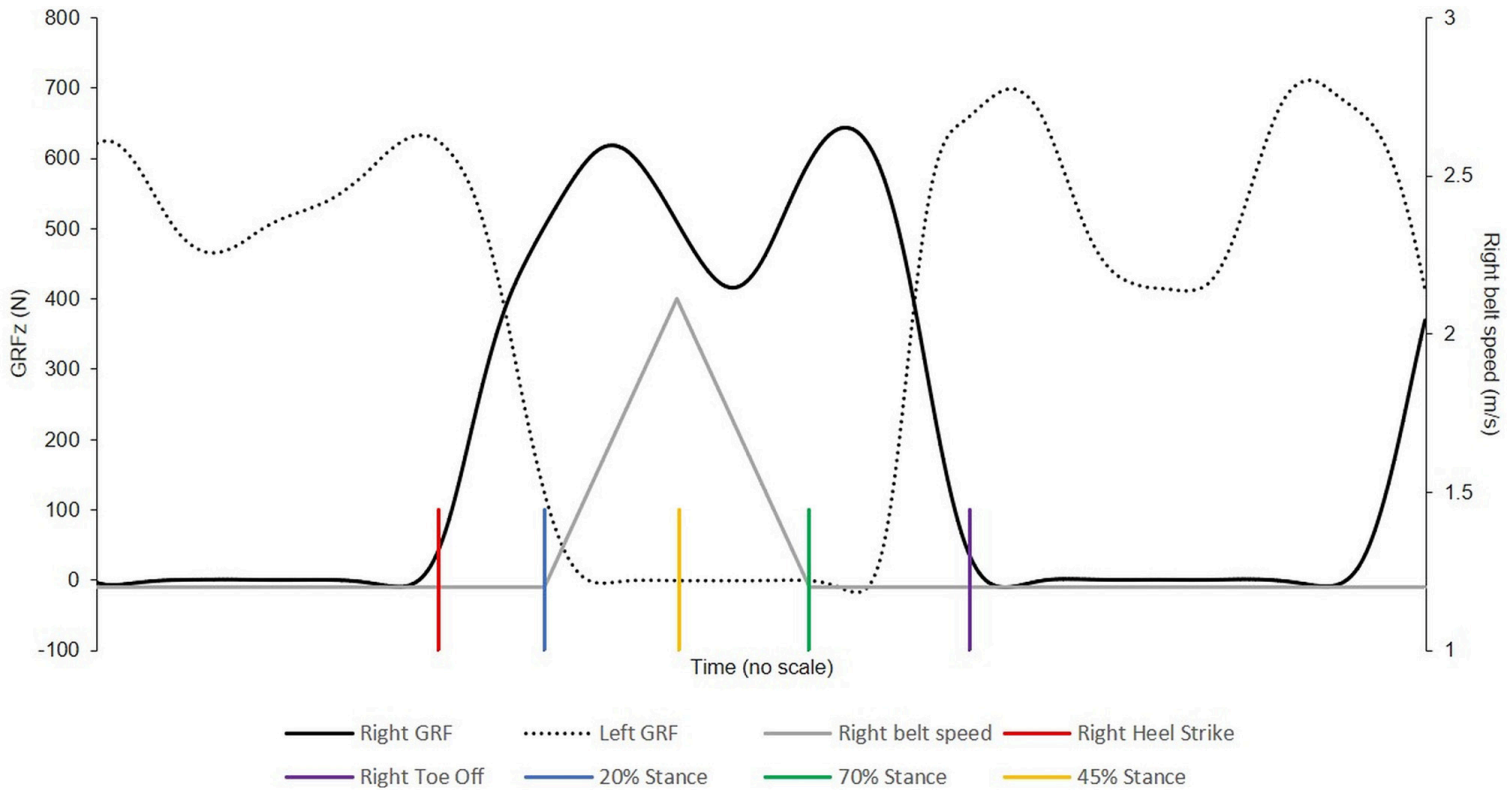
Slip timing representation; the right belt (belt speed represented as the grey line) starts accelerating at 20% of the right leg stance phase and is back to normal (1.2 m·s^−1^) at 70% of the right leg stance phase. GRFz, vertical ground reaction force.

On the basis of regression equations from Kirtley et al. (1985) and pilot data, we estimated that the stance phase of normal walk at 1.2 m·s^−1^ would last ~600 ms. Therefore, to reach a reasonably high maximal belt speed [~2 m·s^−1^, Nagano et al. (2013) reported backward toe sliding velocity during FFSs of ~1.6 (±~1.0) m·s^−1^], we set up the acceleration and deceleration phases to each last 25% of normal stance. We thus aimed for a total peak belt velocity 0.75 m·s^−1^, greater than the initial speed and reaching a peak at 1.95 m·s^−1^. The beginning and end of the acceleration and deceleration phases were triggered at 20 and 70% of stance, respectively, and peak speed reached at approximately 45% of normal stance, which coincided with the beginning of the propulsive phase of gait and increased the anterior acceleration of the body CoM (Figure 1).

By starting the belt posterior acceleration during the single support phase, participants had enough time to contact and land on the ipsilateral leg before the slip and did not have the possibility to compensate with the contralateral leg. By returning to normal speed at about 70% of stance, participants could safely recover if they failed to land on the contralateral belt on the first recovery step. Because the slip reduced the stance phase duration compared with normal condition, the end of the deceleration phase was delayed by about 10% during the slip.

##### Individual adjustments and data collection

During testing, the timing of the slip was individualised according to the Normal stance time of each participant, defined with a 40-N threshold for heel strike and toe-off. This threshold was used to ensure that the application would not trigger the belt acceleration outside of the targeted time frame (e.g., due to a noisy raw force signal during swing or from slight contact from the sole on the treadmill during the swing phase). The application was then manually adjusted so that the delay between heel strike and the beginning of the belt acceleration, as well as the duration of the belt acceleration, were adapted to participant's normative data (stance phase duration in normal conditions).

Following the familiarisation period, participants were informed that we would trigger a gait perturbation at some time while they continued walking on the treadmill. They were not given any indication about what the perturbation would be. We asked them to not use the handrails. Only one perturbation per participant was triggered here, as it is known that participants quickly improve their balance recovery when exposed to repeated perturbations (Bhatt et al., 2006b; Epro et al., 2018b).

#### Protocol Evaluation

As stated above, an ecologically valid FFS would be unexpected, would increase the foot sliding velocity during the perturbed stance phase (posteriorly) and would generate a forward loss of balance. For the application to be experimentally valid, the slip had to be triggered with consistent timing across all participants and allow biomechanical comparisons between individuals.

##### Ecological validity

Kinematic and kinetic data from Normal and Pre1 were used to test whether the slip was unexpected. We thus compared MoS at heel strike (MoS_HS_) and joint moments at the hip, knee and ankle between Pre1 and Normal. Internal joint moments at the hips, knees, and ankles were computed on Visual3d (C-Motion, Germantown, USA) from kinematics and force plate data using inverse dynamics. The slip was considered unexpected when there were no significant differences between Pre1 and Normal.

In this study, MoS_HS_ was measured as the distance between the anterior boundary of the BoS (defined as the distance between the feet second toe-marker positions) and XCoM. Therefore, if the XCoM is positioned anteriorly to the BoS forward boundary, the MoS would be negative and would characterise an unbalanced state:

(1) $$\begin{array}{r} XCoM=P_{CoM}+\frac{V_{CoM}+{\bar{V}}_{foot}}{\sqrt{\frac{g}{L}}} \end{array}$$

where *P*_*CoM*_ is the position of the CoM relative to the second toe marker position of the trailing foot, *V*_*CoM*_ is the velocity of the CoM$,\;{\bar{V}}_{foot}$ is the velocity of the toe marker (averaged during stance phase), *g* is the gravitational acceleration (9.81 m·s^−2^) and *L* is the sagittal distance between the CoM and the ankle joint centre. Segment masses and centres of mass locations were calculated based on Dempster's regression equations, the position of the whole-body CoM as the weighted sum of the 15 body segments.

To quantify whether the perturbation increased foot velocity during the stance phase of Slip, foot's CoM velocity (time derivative of the foot CoM position) was calculated from the kinematic data collected during Slip and compared with Normal.

To confirm whether there was a forward loss of balance during the stance phase of Slip, the instantaneous MoS (MoS_Inst_), as opposed to MoS_HS_, was measured throughout stance. Here, to take into account the effect of the increased belt speed on the foot velocity, ${\bar{V}}_{foot}$ in Equation (1) was replaced by *V*_*foot*_, which was the instantaneous velocity of the toe marker during the stance phase of Slip and compared with MoS_Inst_ calculated during Normal. Consequently, at heel strike of Slip (before the perturbation onset), the scale of MoS_Inst_ was larger than measured in Normal using Equation (1) (MoS_HS_).

##### Experimental validity

Change in belt speed was recorded on D-Flow and normalised to stance duration to evaluate the repeatability of the perturbation timing. Change in MoS_Inst_ was used to evaluate the induced instability timing. We evaluated the perturbation timing and instability timing consistencies by measuring the coefficient of variation (CV) between participants.

### Assessment of Forward Falling Slip Recovery Mechanics

Kinematic data were used to evaluate the dynamic stability during and while recovering from the FFS. We measured MoS_HS_ and BoS size of every recovery step using Equation (1). To quantify how long it took to recover from the perturbation, the number of steps required to return MoS_HS_ back within normal ranges (one standard deviation from mean MoS_HS_ during Normal) for one and three consecutive steps (nsteps_1 and nsteps_3, respectively) were measured.

Kinetic data were used to determine the biomechanical strategies of recovery from the slip. The internal joint moments and GRF vector angle were assessed during Slip and until Rec4 and were compared with Normal. GRF angle to the vertical was measured in the sagittal plane as the inverse tangent of the ratio between the anterior–posterior and vertical components of the GRF, where positive angles reflected an anteriorly oriented GRF.

### Statistical Analysis

For each test, we checked the normality distribution of the data before running the appropriate parametric or non-parametric test.

To validate the first criterion set for simulating a realistic FFS, that is, triggering an unexpected perturbation, we used zero-dimensional paired sample *t*-tests and one-dimensional [statistical parametric mapping (SPM)] paired sample *t*-tests to test whether MoS_HS_ and joint moments were different between Normal and Pre1, respectively. Despite running multiple comparisons, the significance level was kept at *p* < 0.05 between Normal and Pre1, as we wanted to maximise sensitivity to detect even small anticipatory changes that may not have been identified with a lower threshold.

To validate the second and third criteria of realistic FFSs, that is, to increase the foot velocity and create a forward loss of balance during the slip, we used one-dimensional (SPM) paired sample *t*-tests to compare foot's CoM velocity and MoS_Inst_ between Normal and Slip. Here, the significance level was corrected and reached *p* < 0.025 to increase our certainty of detecting meaningful differences. We used a Bonferroni correction as a conservative correction and to minimise the risk of detecting false-positive effects and overinterpreting our data when quantifying the effect of the perturbation on either Slip or recovery steps.

To assess how participants recovered from the slip, we tested whether dynamic stability (MoS_HS_) during Rec1 to Rec15 differed from Normal, using the Friedman test followed by Wilcoxon signed-rank tests with Bonferroni adjustments. As we performed 15 *post hoc* tests, significance was reached for *p* < 0.0033.

To assess whether participants changed the size of their BoS between Normal and the first four recovery steps, we used repeated measures one-way ANOVA with Bonferroni adjustments. We compared joint moments between Slip, Rec1 to Rec4 and Normal using non-parametric equivalent of repeated measures ANOVA on SPM followed by *post hoc* tests with Bonferroni adjustments; here, significance was reached at *p* < 0.0102. We compared the time at which the GRF angle became anterior between Normal and Slip, and between Normal and Rec1 by using the Friedman test and the Wilcoxon signed-rank tests with Bonferroni adjustments. As we performed five *post hoc* tests, significance was reached for *p* < 0.01.

Data are presented as mean (±SD) along with 95% confidence intervals (CIs) where appropriate, unless otherwise stated. We used SPSS 26 (IBM, NY) for 0D measurements (MoS_HS_ and BoS) and SPM for 1D measurements (every other variable including MoS_Inst_) on Matlab (Mathworks, R2018).

## Results

### Slipping Perturbation Protocol

**Figure 2:**
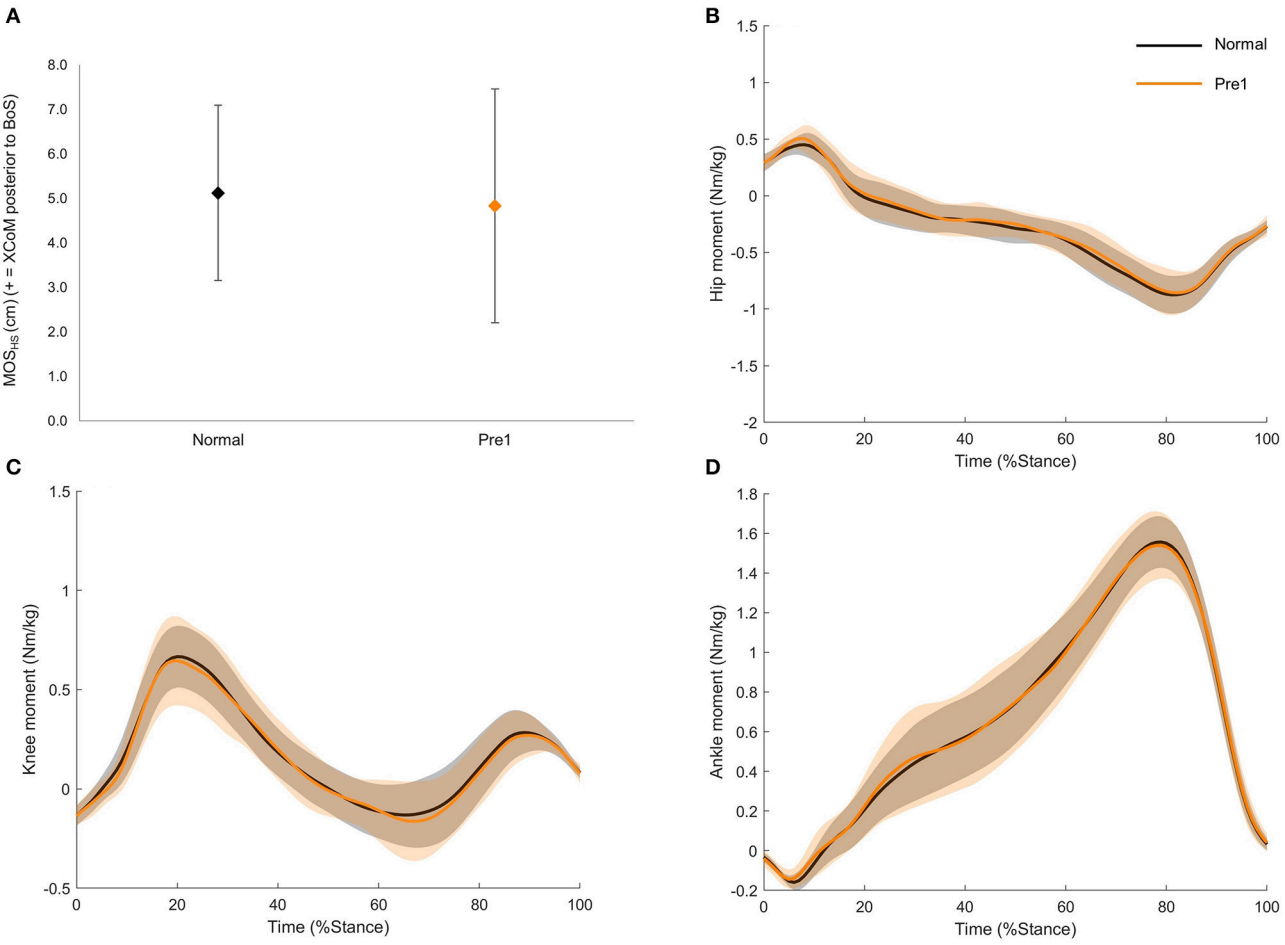
**(A)** Group mean and standard deviation of the margin of stability at heel strike (MOS_HS_) for Normal and Pre1. **(B)** Group mean and standard deviation (shaded region) hip moment for Normal and Pre1. **(C)** Group mean and standard deviation (shaded region) knee moment for Normal and Pre1. **(D)** Group mean and standard deviation (shaded region) ankle moment for Normal and Pre1. Positive moments are extensors, and negative are flexors. Black diamond and lines represent Normal; orange diamond and lines represent Pre1. No significant differences were detected for any parameter.

There were no significant differences (*p* > 0.05) between Normal and Pre1 for MoS_HS_ or joint moments (hip, knee and ankle), indicating no pre-adaptations prior to the perturbation (Figure 2).

**Figure 3:**
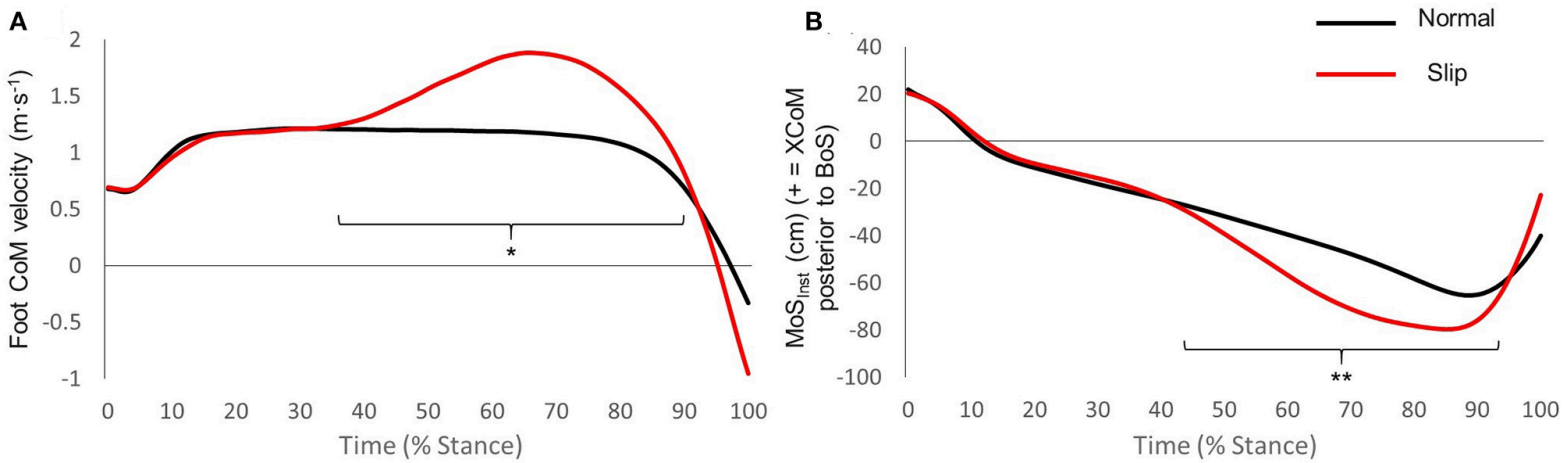
**(A)** Group mean foot centre of mass velocity for Normal and Slip. *Significantly different from Normal, *p* = 0.010. **(B)** Group mean instantaneous margin of stability (MoS_Inst_) for Normal and Slip. **Significantly different from Normal, *p* < 0.001. Black lines represent Normal, and red lines represent Slip.

The foot's CoM velocity during Slip was significantly higher than that during Normal (*p* = 0.010) from 34 to 90% of stance, and MoS_Inst_ was significantly lower than Normal from 43 to 93% of stance, indicating that the XCoM was further ahead of the anterior boundary of the BoS (Figure 3), with the peak instability occurring at 84.8 (±2.6)% of stance (95% CI: 83.5 to 86.1).

For 12 of the participants, the belt speed increased at 24.9 (±1.3)% of stance (95% CI: 24.1 to 25.7) and was back to normal (1.2 m·s^−1^) at 86.3 (±3.5)% of stance (95% CI: 84.1 to 88.5), with a very good consistency for both timing of the perturbation and timing of peak instability (CV = 5% and 3%, respectively), indicating that the perturbation was experimentally valid. Isolated technical difficulties with the sampling frequency on D-Flow prevented this calculation for the remaining participants.

### Assessment of Forward Falling Slip Recovery Mechanics

#### Dynamic Balance

**Figure 4:**
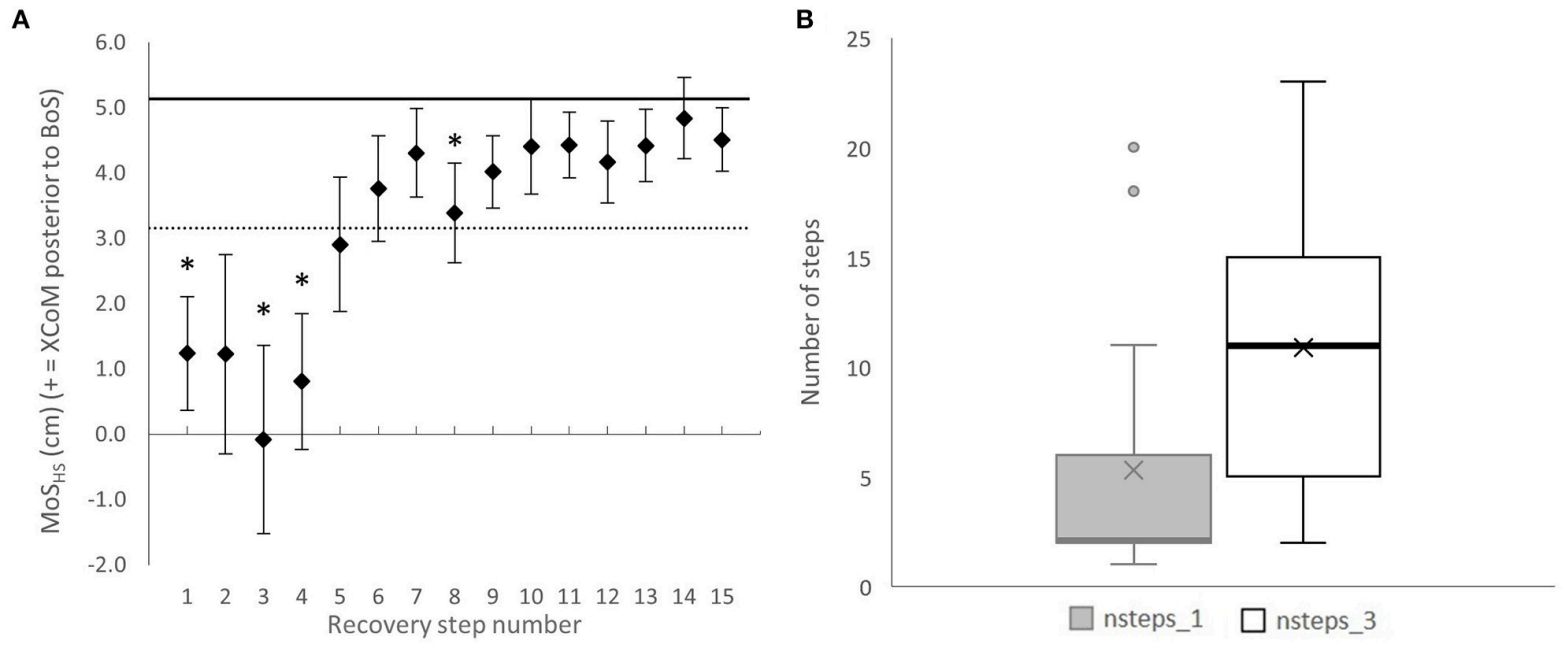
**(A)** Group mean margin of stability at heel strike (MoS_HS_) from the first to 15th recovery step with standard error. The solid horizontal black line represents the averaged MoS_HS_ during Normal gait; the dotted horizontal black line represents one standard deviation from Normal. *Significantly different from Normal, *p* < 0.0033. **(B)** Box plot representing the number of steps required to have one step back within normal range (nsteps_1, grey box) and the number of steps required to have at least three consecutive steps back within normal range (nsteps_3, white box). The lower (Q1) and upper quartiles (Q3) represent observations outside the 25–75th percentile range. The diagram shows the mean (crosses) and median (thick horizontal lines) for nsteps_1 and nsteps_3, respectively (for nsteps_1: median = Q1; therefore, the line of nsteps_1 overlays the one of Q1). Data falling outside Q1 and Q3 are outliers.

MoS_HS_, which represents the dynamic balance, was significantly lower in Rec1, Rec3, Rec4, and Rec8 than in Normal (*p* < 0.0033) (Figure 4), meaning that the distance between the XCoM and the anterior boundary of the BoS was reduced. Participants needed 5.3 (±5.8) steps (95% CI: 2.3 to 8.3) to have at least one step back within 1SD of Normal MoS_HS_, and 10.9 (±7.0) steps (95% CI: 7.3 to 14.5) to have at least three consecutive steps back to Normal MoS_HS_ (Figure 4).

#### Recovery Strategy

**Figure 5:**
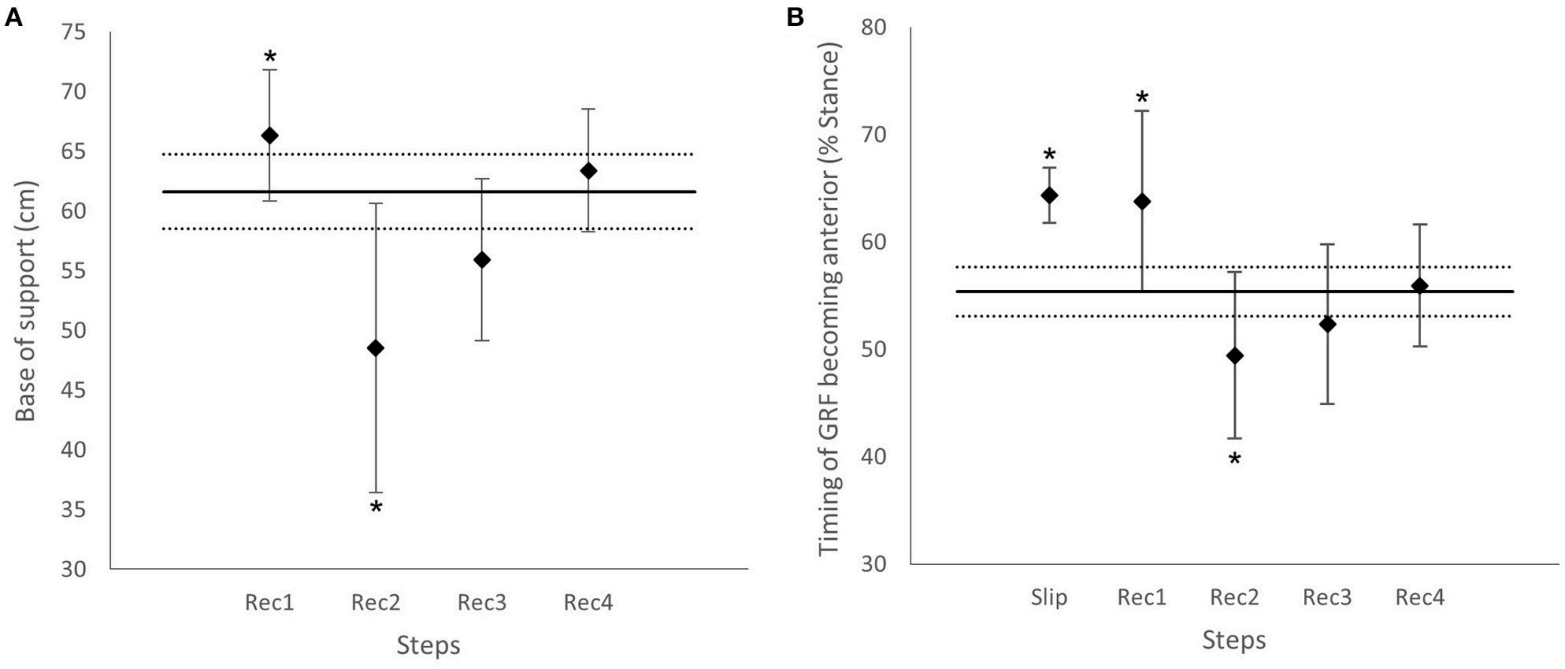
**(A)** Group mean base of support at heel strike (BoS) from the first to fourth recovery steps with standard deviation. The solid horizontal black line represents the averaged BoS during Normal gait; the dotted horizontal black lines represent one standard deviation from Normal. *Significantly different from Normal, *p* < 0.01. **(B)** Group mean timing of ground reaction force (GRF) angle becoming anterior from Slip to Rec4, with standard deviation. The solid horizontal black line represents the averaged GRF angle becoming anterior during Normal gait; the dotted horizontal black lines represent one standard deviation from Normal. *Significantly different from Normal, *p* < 0.01.

**Figure 6:**
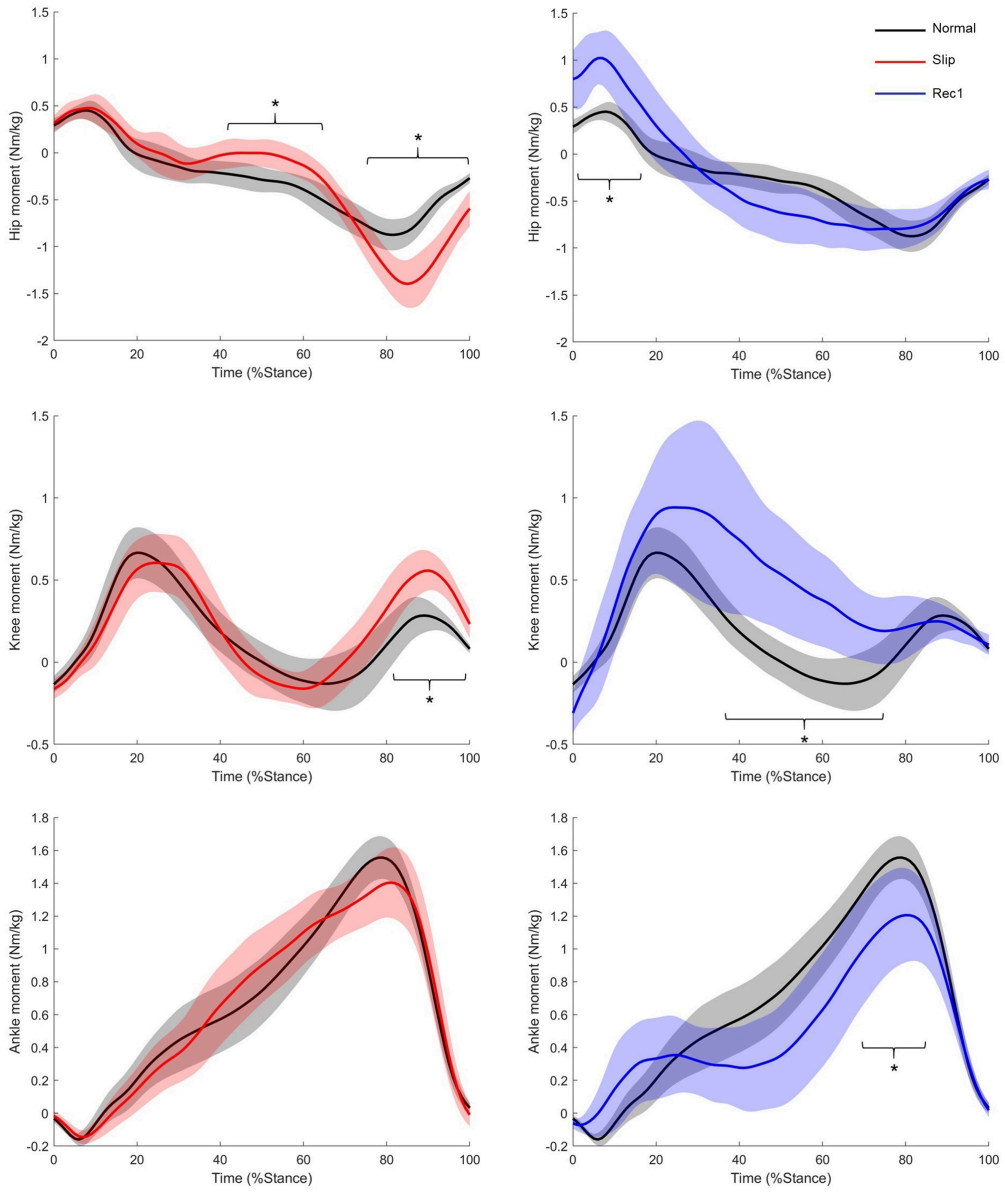
Hip, knee, and ankle moments during Normal (black lines, SD shaded regions), Slip (red lines, SD shaded regions) and first recovery step (blue lines, SD shaded regions). Positive moments are extensors. *Significantly different from Normal: *p* < 0.001.

**Figure 7:**
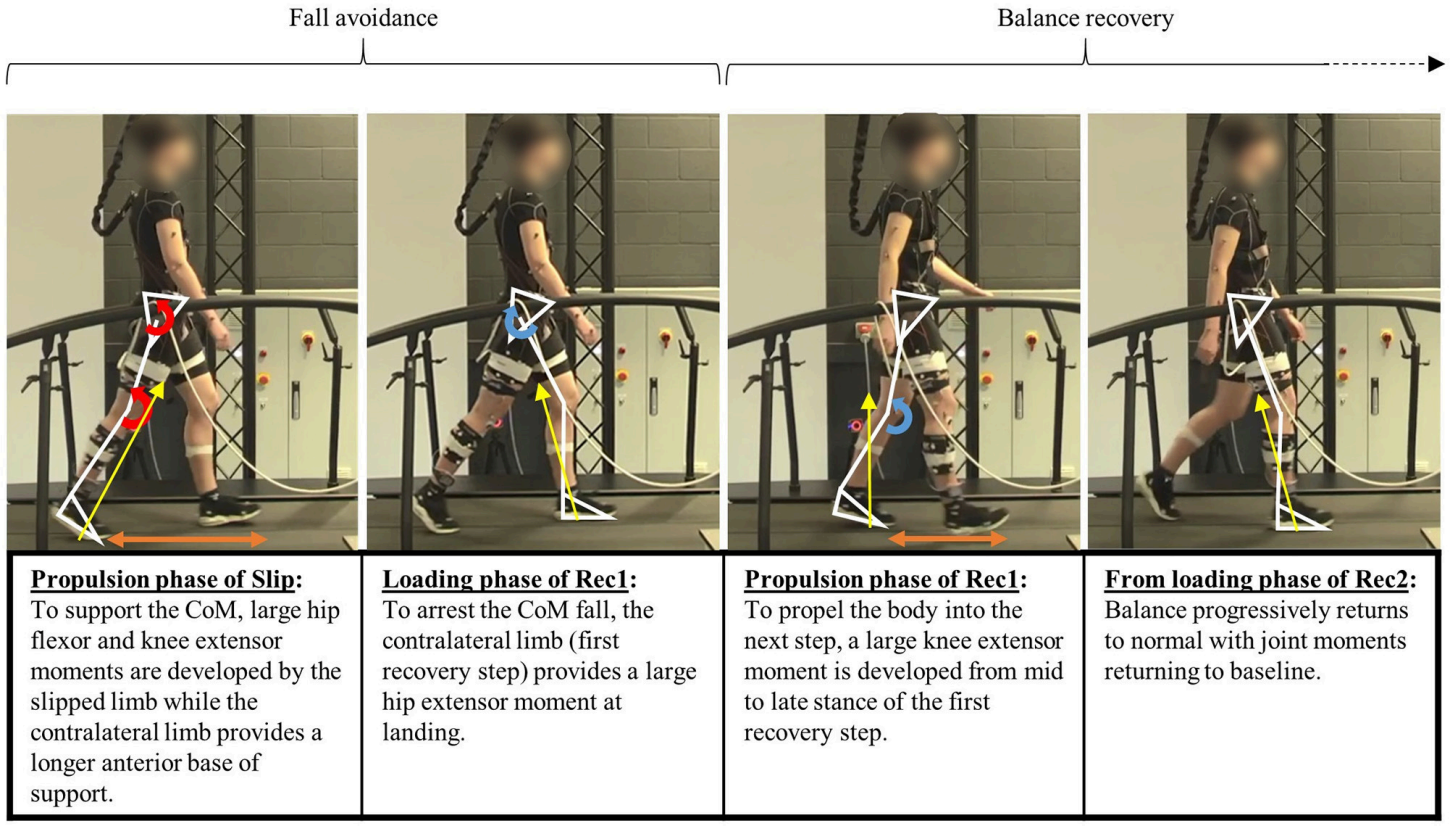
Fall avoidance and balance recovery sequences. Red curved arrows represent joint moments significantly different from Normal during Slip (*p* < 0.001), blue curved arrows represent joint moments significantly different from Normal during Rec1 (*p* < 0.001) and orange arrows represent base of support significantly different from Normal (*p* < 0.01). Yellow arrows represent the direction of the ground reaction force (GRF) vector.

Distinct phases of the slip and first recovery steps were apparent. After the onset of the belt acceleration, Slip could be divided into perturbation (~20 to 70% stance) and propulsion (~70 to 100% stance) phases. Rec1 and the following recovery steps were subdivided into loading phase (~0 to 20% stance) and midstance–propulsion phase (~20 to 100% stance). The biomechanical data associated with each of these phases are described below (Figures 5–7).

##### During the slip

During the perturbation phase of Slip, the GRF vector stayed posterior longer, crossing the vertical later than in Normal (*p* < 0.001, Figure 5), and the hip flexor moment decreased, acting like a passive joint (*p* < 0.001, Figure 6).

During the propulsion phase of Slip, the GRF angle was greater anteriorly than Normal (*p* < 0.001, Supplementary Material), and the hip and knee joints generated higher flexor and extensor moments, respectively (*p* < 0.001, Figure 6). There was no significant difference in ankle moments between Normal and Slip (*p* > 0.0102). The BoS at heel strike of Rec1 was significantly longer than in Normal (*p* < 0.001, Figure 5).

##### During the first recovery step

During the loading phase of Rec1, participants restrained the rotation of the body by generating a larger hip extensor moment than in Normal (*p* < 0.001, Figure 6), and the GRF became anterior later than in Normal (*p* = 0.003, Figure 5).

During the midstance–propulsion phase of Rec1, propulsion mainly came from a large knee extensor moment (*p* < 0.001, Figure 6), whereas the plantarflexor moment was significantly lower than in Normal (*p* < 0.001, Figure 6). The BoS at heel strike of Rec2 was shorter than in Normal (*p* = 0.006, Figure 5).

Complete GRF vector angle data are presented in the Supplementary Material.

##### On the remaining steps

The remaining recovery steps progressively returned to Normal until Rec4, where there were no differences in GRF angle and joint moments compared with those in Normal (data for all steps are presented in the Supplementary Material).

## Discussion

This study successfully designed and applied a simulated FFS on an instrumented treadmill using a semi-automatic application. The perturbation triggered in the present study met our criteria to be defined as ecologically valid (i.e., no pre-adaptation, increased foot posterior velocity and forward loss of balance during the slip) and experimentally valid (i.e., repeatable and adjustable). Using this protocol, we successfully studied the recovery mechanics from an FFS for the first time.

After the slip, participants needed on average 11 steps to restore balance back to normal for at least three consecutive steps. This recovery (Figure 4) was not as linear and required more steps to return to baseline MoS_HS_ than previously reported in trips (Suptitz et al., 2013). This could result from a larger effect on the whole-body angular momentum during slips than trips. Indeed, the angular momentum of the body can be measured as the sum of segment angular momenta around the CoM (Herr and Popovic, 2008). Thus, theoretically, by increasing the displacement and velocity of the ipsilateral lower limb segments during a slip (instead of momentarily decreasing them during a trip), the same perturbation intensity (that could be indirectly evaluated by measuring the change in instantaneous MoS) would result in a larger angular momentum and a more challenging balance control. This argument is supported by findings from Yoo et al. (2019) showing that hip, knee and ankle moments generated during the first recovery step following a BFS were larger than those generated when recovering from trips, suggesting that slips recovery may be more demanding than trip recovery.

Another possible explanation to the larger number of steps required to recover from FFSs may lay in the control of the BoS, which seems to differ depending on the type of perturbation triggered. Following a trip, when landing on the perturbed leg (equivalent to Rec1 in the present paper), the BoS shortened compared with that in normal, before lengthening on the second heel strike following the trip and finally being back to normal range from the third recovery step (Suptitz et al., 2013; Epro et al., 2018a). These results, together with those from Karamanidis et al. (2008), suggest that balance recovery from forward falling perturbations mainly relies on an increased size of the BoS. In the present study, however, we found that whereas on the first recovery step participants increased their BoS, the MoS was lower than in Normal, and that whereas on the third and fourth recovery steps the BoS was not significantly different from the Normal anymore, the MoS was significantly smaller than in Normal. This suggests that balance recovery following FFSs could depend on both the control of the state of the CoM and the control of the BoS size, as previously showed by Bhatt et al. (2005) on BFSs.

Results from the present study on the MoS size reduction for the first recovery steps following the perturbation are contradictory to those reported in previous studies inducing FFS-like perturbations (Madehkhaksar et al., 2018; Roeles et al., 2018), which found no significant change in the size of the MoS. These discrepancies are likely due to methodological differences with the authors reporting the MoS on an average of multiple steps, and on the presence of a familiarisation trial (Madehkhaksar et al., 2018), which may have triggered a learning effect and improved the balance recovery following the perturbation.

During the slip, the only kinetic adaptation we detected was a reduced hip flexor moment of the slipped limb, which approached 0 N·m/kg. It is likely that the lack of instantaneous active response allows for passive hip extension and to not impose a large angular acceleration on the trunk. Therefore, this may be a strategy to minimise the destabilising effect of the FFSs.

According to the kinetic data in this study, we conceptualised the mechanics of the recovery after the FFSs as being made up of four different phases (Figure 7). Immediately following the slip, participants relied on large hip flexor and knee extensor moments (respectively 1.6 and 1.9 times greater than in Normal) from the slipped limb to support the CoM and allow time for the contralateral limb swing to provide a more anterior BoS, which was longer than that in Normal. The second phase corresponded to loading of the contralateral limb (Rec1) to “catch” the body and arrest the fall with an increased hip extensor moment (2.4 times higher than in Normal). These first two phases were directed at preventing the slip from becoming a fall. Subsequently, the propulsive phase of the contralateral limb (Rec1) utilised greater knee extensor moments (averaged 3.2 times greater than in Normal) to raise and advance the CoM into the next step, which was shorter than Normal. The final stage of recovery was made up of multiple steps with progressively more normal mechanics to restore typical balance. The kinetic strategies used in each phase are visualised in Figure 7.

To our knowledge, recovery and joint moments during or after FFSs have not been reported previously, so we can only compare the strategies we document here with those of trips and BFSs, keeping in mind that the dynamic conditions leading to these perturbations are completely different. In BFSs, whereas small increases in hip flexor moments were reported from 40 to 55% of stance (Cham and Redfern, 2001), larger knee extensor moments were clearly reported during recovery (Liu and Lockhart, 2009; Yoo et al., 2019) and were considered critical in the sagittal balance recovery following a slip. The greater hip extensor moment that we identify as important in arresting the fall early in Rec1 and the larger propulsive knee extensor moment that follows were also identified in the first step after trips with elevating recovery strategies (King et al., 2019). However, they are inconsistent with the recovery kinetics reported by Yoo et al. (2019), who caused a forward fall by using a treadmill to simulate a trip in early stance. Taken together, the discrepancies between studies indicate that the timing and mechanics of how the simulated perturbation is applied are important in determining the recovery strategy. Nonetheless, similar to findings from most trips and BFSs studies, hip and knee joint kinetics therefore play a crucial role in fall avoidance and balance recovery during FFSs and highlight the need to target their strength in fall-prevention programs.

We did not find such an important role of muscles around the ankle joint though, with a peak moment being 1.3 times larger in Normal than in Rec1. In trips, increased plantarflexor moments during the push-off phase of the contralateral leg were found (King et al., 2019), and deficits in plantarflexor moments were linked to decreased recovery capacities in older adults (Pijnappels et al., 2005). In BFSs, however, results reported on ankle moments were contradictory, with some authors reporting diminished plantarflexor moments (Cham and Redfern, 2001), some reporting increased dorsiflexor moments (Liu and Lockhart, 2009) and others reporting no significant changes in either direction (Yoo et al., 2019). In the present study, we did not find any significant contribution of the ankle during fall avoidance, and we only found a lower plantarflexor moment during the propulsion phase of Rec1, indicating that the ankle may have a less dominant role in fall arrest and balance recovery from an FFS than the hip and knee.

These will have implications in the development of specific training protocols. Considering the increased plantarflexor moments developed while recovering from trips, training interventions targeting specifically the triceps surae muscles have been developed (Epro et al., 2018b). Although they successfully improved the muscle function, they did not improve balance recovery from a trip. Quadriceps strength training however was found to be linked with decreased risks of falling in older adults (Day et al., 2002; LaStayo et al., 2003). If the results of the present study are similar in older adults, quadriceps strengthening should be implemented in interventions aiming to decrease falls induced by slips. Skills training have also been successfully tested in older adults for both BFSs and trips, with participants improving their balance recovery with repeated perturbations and retaining the effect of the training on long period (Bhatt et al., 2006a; Epro et al., 2018b). These improvements were attributed to neural adaptations and the creation of new motor programs within the central nervous system.

In this first study reporting and trialling our protocol to simulate FFSs, there were some limitations, which should be acknowledged. Owing to some investigator errors in the semi-automatic application during data collection, we had to remove two participants from the sample. A fully automatised application would be better adapted to the protocol and would avoid losing data. Despite offering the opportunity to collect more data than a walkway, the treadmill also comes with some limitations. Firstly, the slipping of the foot during stance was limited to the sagittal plane, whereas new evidence suggests that the displacement of the foot during FFSs is both posterior and lateral (Rasmussen and Hunt, 2019). Secondly, our preliminary results also suggest a larger lateral sway of the body CoM than Normal during the first recovery step, which has to be compensated to avoid lateral loss of balance. As walking on a split-belt treadmill increases the width of the BoS (Zeni and Higginson, 2010), it is possible that this kind of treadmill facilitates the frontal balance recovery compared with overground walking. Despite these, we managed to create a protocol that simulates an FFS, which met our criteria for being ecologically and experimentally valid to study the recovery strategies across as many steps as required. Future work should seek to understand whether this treadmill protocol can be used to quantify the medio-lateral forces and motion of an FFS also.

Further, all our participants managed to recover from the perturbation (i.e., mechanical fall arrest system was never triggered). We do not have normative data from actual slips to compare the velocity and intensity of the perturbation with those in real environments. Although we reached a larger toe sliding velocity (1.9 ± 0.1 m·s^−1^) than the average (~1.6 ± 1.0 m·s^−1^) reported by Nagano et al. (2013) with participants slipping on an oily surface in a lab-based experiment, the averaged MoS_HS_ measured in the present study remained positive following the slip (Figure 3). It is then possible that the perturbation triggered here was too light to reflect authentic dangerous situations especially in a healthy population. Triggering several perturbations with different belt accelerations until participants actually fell would have been a solution to adapt the slip intensity to individual abilities. However, we know from the literature on trips (Epro et al., 2018b) and BFSs (Bhatt et al., 2006a; Pai et al., 2014a,b) that a learning effect occurs when participants are exposed to repeated perturbations. This is likely to be the case for FFSs too; therefore, this approach would have jeopardised our efforts to produce a database for balance recovery following the first instance of a slip. For the purpose of this study, however, perturbations were intentionally not strong enough to cause a fall, as we aimed to understand the mechanisms of recovery.

We found a large variability between participants' response to the perturbation (Figure 4A), which could be due to the imposed walking speed used, as changes in walking speed by more or <20% from comfortable speed were found to change recovery strategies following a trip (Krasovsky et al., 2014). By using a predefined walking speed (as opposed to self-selected), we do not take into account individuals' capacity. It is possible that the challenge imposed by a perturbation triggered at 1.2 m·s^−1^ may be dependent on factors such as age and physical activity level. However, as the MoS is directly dependent on the walking speed, comparing the dynamic balance of participants walking at different speeds would not have been possible. In a recent paper, McCrum et al. (2019) suggested that walking speed should be adapted to each participant to reach a similar MoS for all of them. This should decrease the MoS variability between participants and allow a better measurement of the perturbation intensity and of the dynamic adjustments made to return to normal balance.

To conclude, this study was the first one to quantify the kinetic requirements of balance recovery following an FFS, meeting the criteria we defined for an ecologically and experimentally valid perturbation. We found that recovery requires the development of large hip and knee internal moments, which may be problematic for older adults with diminished muscle strength. Therefore, we will next trial the same protocol with older adults to understand whether and how (1) they recover from an FFS, (2) their performance is conditioned by their muscle strength and (3) we can decrease their risks of falling with an adapted training protocol.

## Data Availability Statement

The datasets generated for this study are available on request to the corresponding author.

## Ethics Statement

The studies involving human participants were reviewed and approved by Liverpool John Moores University Ethics Committee. The patients/participants provided their written informed consent to participate in this study.

## Author Contributions

HD, TO'B, and CM: contributed to the conception and design of the research. HD, CH-A, and KH: data acquisition. HD: data analysis. HD, TO'B, and CM: interpretation of the results. HD: drafted the manuscript. All authors edited and revised the manuscript. All authors agreed to the manuscript submission for publication.

## Conflict of Interest

The authors declare that this study received funding from Minerva Research Labs Ltd. (London, UK). The funder was not involved in the study design, collection, analysis, interpretation of data, the writing of this article or the decision to submit it for publication.
