# Supplementary material for: Recovery From a Forward Falling Slip: Measurement of Dynamic Stability and Strength Requirements Using a Split-Belt Instrumented Treadmill
Source: Front Sports Act Living. 2020 Jul 21;2:82. doi: 10.3389/fspor.2020.00082 (PMC7739594; doi:10.3389/fspor.2020.00082)
Supplement: Supplementary file 1 [file Data_Sheet_1.docx]

Recovery from a forward falling slip: measurement of dynamic stability and strength requirements using a split-belt instrumented treadmill.

Supplementary Material

# Supplementary Data

In the main manuscript, we used zero-dimensional statistical analysis to show that the time at which the GRF vector becomes anterior is different between Normal and Slip, Normal and Rec1, and Normal and Rec2.

We also used one-dimensional non-parametric equivalent of repeated measures ANOVA on SPM followed by post-hoc tests with Bonferroni adjustments (significance was reached for p<0.0102) to compare the GRF angle (deg) between Normal, Slip, Rec1 to Rec4. We found that the GRF vector was more posterior from 24 to 66% of stance and more anterior from 75 to 90% of stance in Slip than in Normal, less posterior from 24 to 50% of stance in Rec2 than in Normal, and less posterior from 10 to 34% of stance in Rec3 than in Normal (Supplementary Material Figure 1).

# Supplementary Figures

#
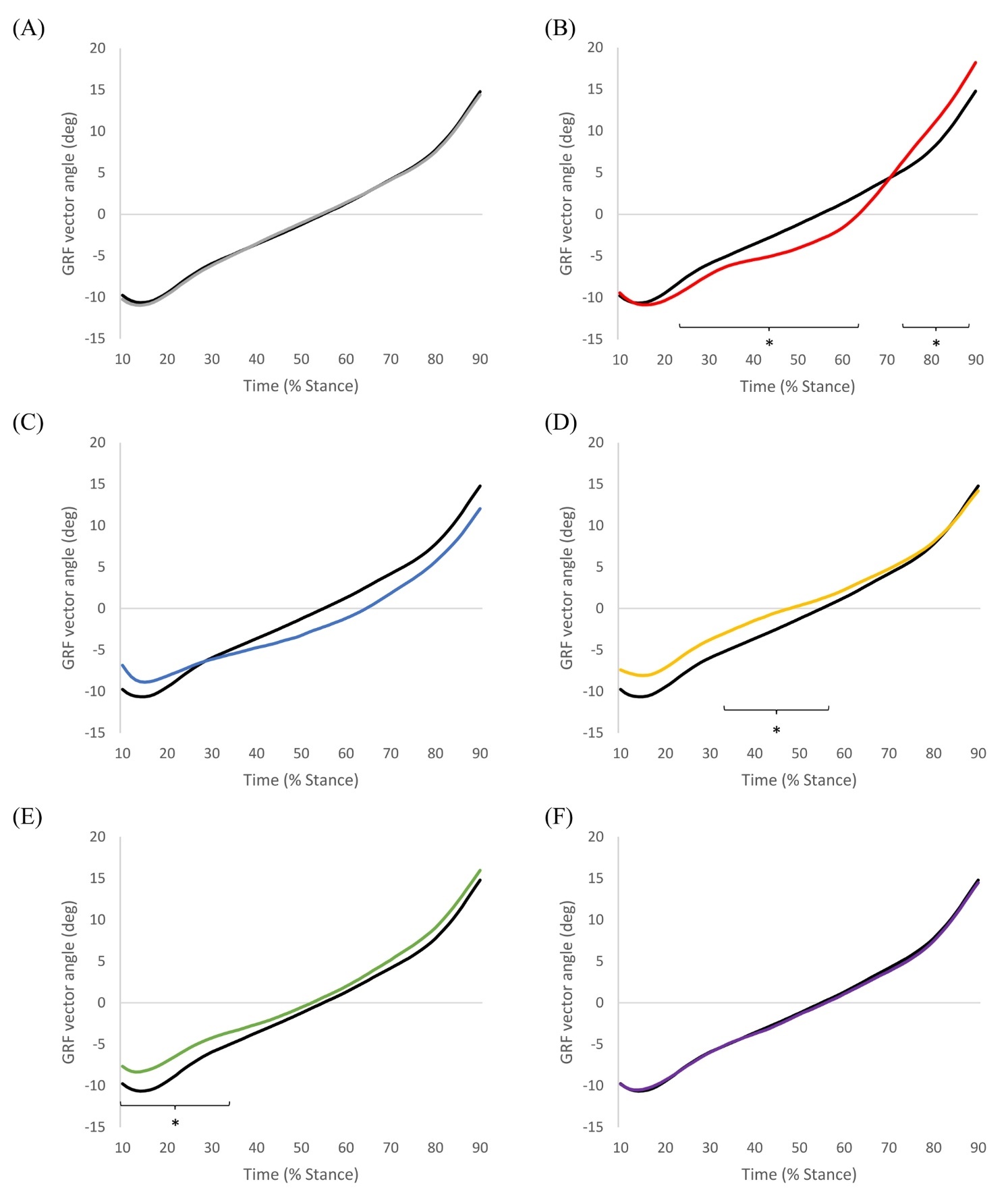
Supplementary Figure 1: (A) Ground reaction force (GRF) vector angle between Normal (black) and Pre (grey), (B) GRF angle between Normal and Slip (red), (C) GRF angle between Normal and Rec1 (blue), (D) GRF angle between Normal and Rec2 (yellow), (E) GRF angle between Normal and Rec3 (green), (F) GRF angle between Normal and Rec4 (purple). Positive is anterior, negative is posterior. * Significantly different from Normal: p<0.001.

**
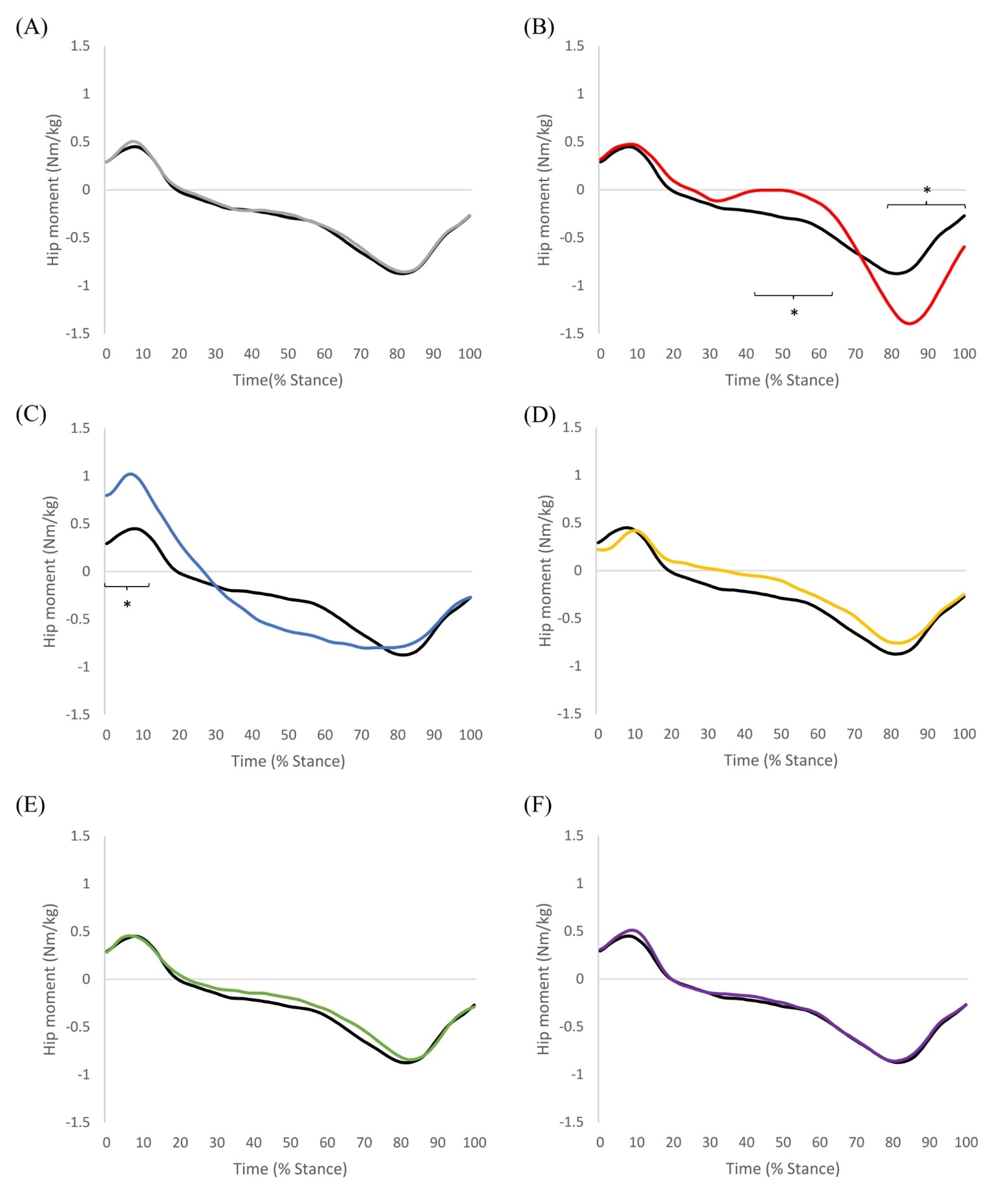
Supplementary Figure 2:** (A) Hip moment between Normal (black) and Pre (grey), (B) Hip moment between Normal and Slip (red), (C) Hip moment between Normal and Rec1 (blue), (D) Hip moment between Normal and Rec2 (yellow), (E) Hip moment between Normal and Rec3 (green), (F) Hip moment between Normal and Rec4 (purple). Positive = extensor. *Significantly different from Normal: p<0.001.


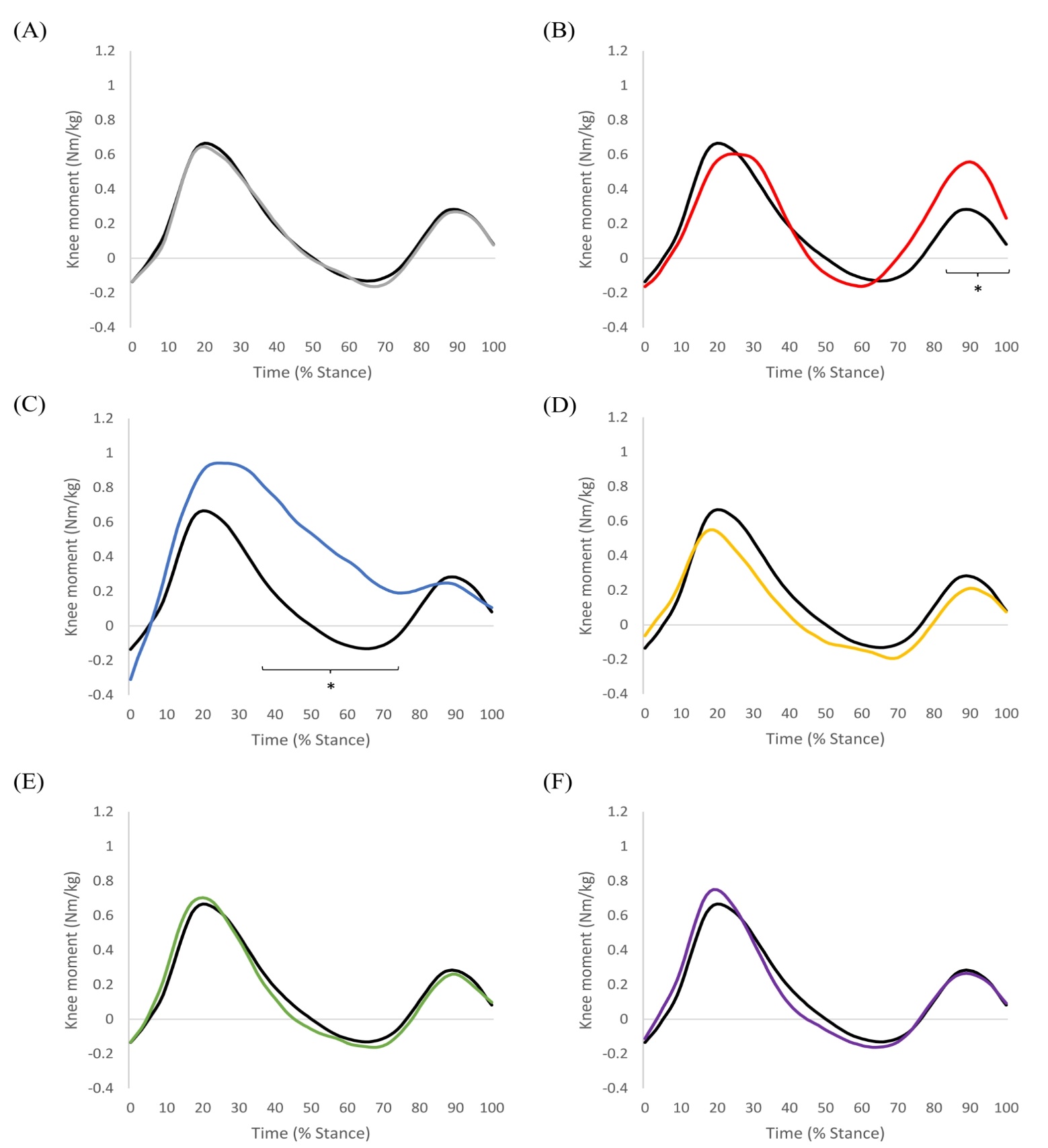


**Supplementary Figure 3:** (A) Knee moment between Normal (black) and Pre (grey), (B) Knee moment between Normal and Slip (red), (C) Knee moment between Normal and Rec1 (blue), (D) Knee moment between Normal and Rec2 (yellow), (E) Knee moment between Normal and Rec3 (green), (F) Knee moment between Normal and Rec4 (purple). Positive = extensor. * Significantly different from Normal: p<0.001.


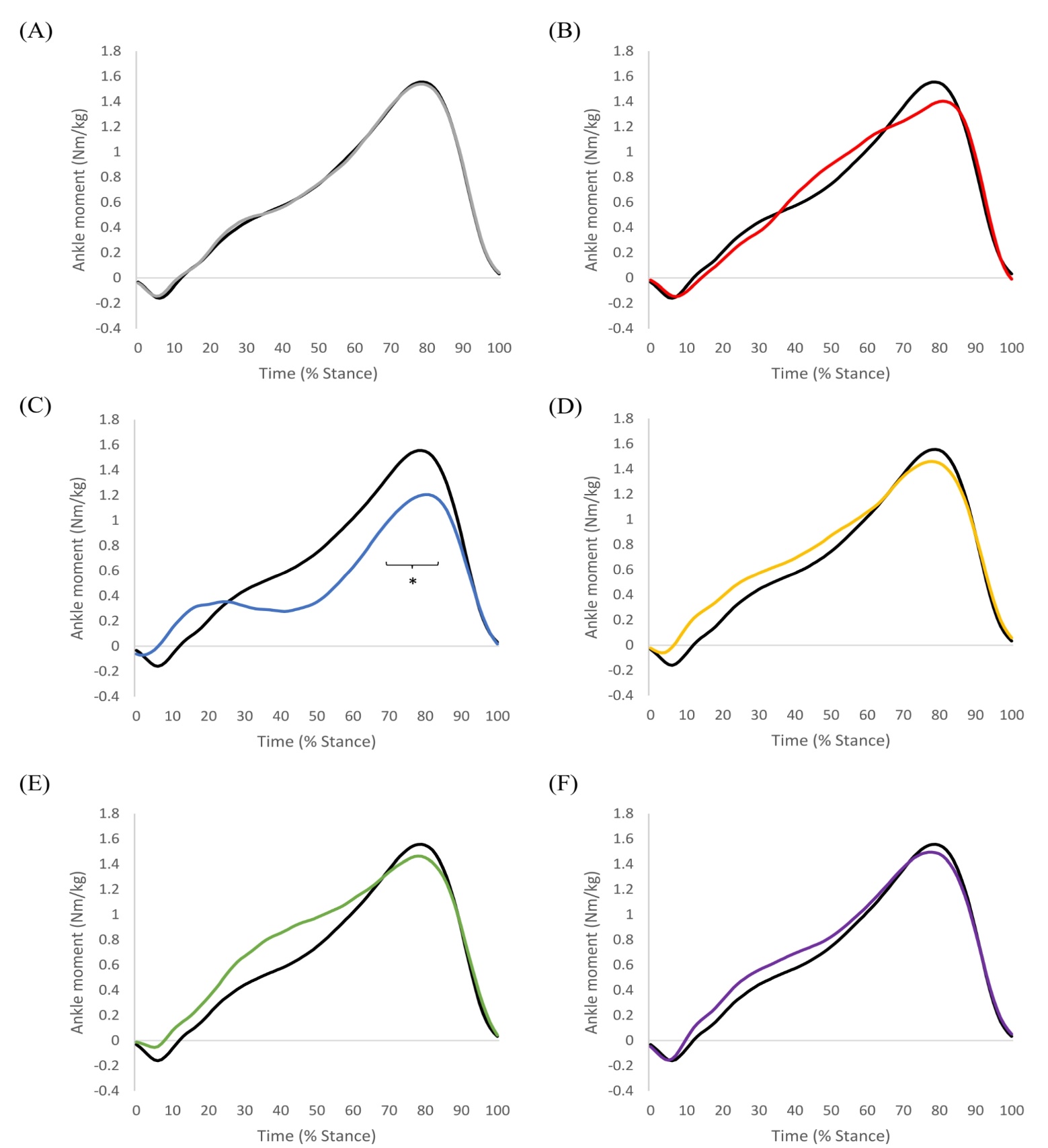


**Supplementary Figure 4:** (A) Ankle moment between Normal (black) and Pre (grey), (B) Ankle moment between Normal and Slip (red), (C) Ankle moment between Normal and Rec1 (blue), (D) Ankle moment between Normal and Rec2 (yellow), (E) Ankle moment between Normal and Rec3 (green), (F) Ankle moment between Normal and Rec4 (purple). Positive = extensor. * Significantly different from Normal: p<0.001.
